# Implication of Leptin-Signaling Proteins and Epstein-Barr Virus in Gastric Carcinomas

**DOI:** 10.1371/journal.pone.0130839

**Published:** 2015-07-06

**Authors:** Euno Choi, Sun-ju Byeon, Soo Hee Kim, Hyun Ju Lee, Hyeong Ju Kwon, HyeSeong Ahn, Dong Ha Kim, Mee Soo Chang

**Affiliations:** 1 Department of Pathology, Seoul National University Boramae Hospital, Seoul National University College of Medicine, Seoul, Korea; 2 Department of Surgery, Seoul National University Boramae Hospital, Seoul, Korea; The University of North Carolina at Chapel Hill, UNITED STATES

## Abstract

We investigated the clinicopathological implications of leptin-signaling proteins and Epstein-Barr virus (EBV)-infection status in gastric carcinomas. Immunohistochemistry for leptin signalling-related proteins (leptin, leptin-receptor, pSTAT3, ERK, pAkt, mTOR and HIF-1 alpha), and *in situ* hybridization for EBV-encoded small RNAs was performed in 343 cases of gastric carcinomas. The siRNA against leptin-receptor was transfected into three stomach cancer cell lines, and western blot for caspase 3 was performed. The TNM stage was a prognostic factor in all 343 patients, and was negatively correlated with expression of leptin, pSTAT3, ERK, pAkt, mTOR and HIF-1 alpha (*P* < 0.05). Leptin-receptor expression was correlated with poor survival in 207 patients of the advanced gastric cancer (AGC) subgroup, 139 of the Lauren diffuse group, and in 160 patients with lymph node metastasis (*P* < 0.05, respectively). Additionally, in stomach cancer cells, cleaved caspase 3 level increased by leptin-receptor inhibition, that is, apoptosis increased. Interestingly, EBV-positive AGC (n = 29) tended to show better survival of patients than EBV-negative AGC (n = 178) (*P* = 0.06). pAkt expression was related with a good survival of 32 patients (9%) in the EBV-positive subgroup, but was not an independent prognostic factor. Among, leptin signaling-related proteins, expressions of leptin-receptor and mTOR were different between EBV-positive subgroup and EBV-negative subgroup (*P* < 0.05, respectively). In conclusion, leptin-signaling proteins and EBV status show different significance on patient survival, according to subsets of gastric carcinomas. The leptin-receptor may predict poor patient prognosis in the AGC, Lauren diffuse and lymph node metastasis subgroups, while EBV-positive status can show a good prognosis in the AGC. Each leptin signaling-related protein may be differently involved in carcinogenesis of EBV-negative and EBV-positive subsets.

## Introduction

Gastric carcinoma is the most common malignancy except for thyroid carcinoma, and ranks as the third cause of cancer death in Korea [1]. The worldwide occurrence of gastric carcinomas is estimated as 8%, and the mortality accounts for 10% of all cancer cases [2]. Despite current advances in the understanding of risk factors associated with gastric carcinoma, its etiology is not fully understood. It is commonly accepted that multiple stepwise molecular alterations contribute to gastric carcinogenesis [3]. Hence, it is important to identify molecular biomarkers that can be used as prognostic factors for effective management of gastric carcinoma patients.

Leptin is a hormone with a molecular mass of 16kDa. It is mainly produced by adipose tissue and secreted into plasma [4], and detected in variable tissues, including placenta, skeletal muscle, ovaries, mammary epithelial cells and the gastric mucosa [5]. Leptin was initially known to play a critical role in controlling food intake and energy balance [4, 6]. Leptin functions *via* the leptin-receptor, a single-transmembrane-domain receptor of the class I cytokine-receptor family, localized to the cell membrane [7]. Recently, it was suggested that leptin and its receptor act as a potential tumorigenic factor by decreasing cell apoptosis, increasing cell proliferation, regulating cell differentiation and promoting angiogenesis in various carcinomas [8–11]. Its tumorigenic action is mediated *via* multiple signaling pathway activation, including JAK/STAT, Raf/MER/extracellular-regulated kinase (ERK), PI3K/Phosphatase and the tensin homolog/Akt/mTOR (PI3K/PTEN/Akt/mTOR) pathways [7, 12, 13]. Leptin expression can be up-regulated through hypoxia-inducible factor-1(HIF-1) alpha, which controls tumor angiogenesis in many solid tumors [8]. Furthermore, leptin-receptor is activated directly by HIF-1 alpha [14]. There have been controversial results with respect to its prognostic effects on gastric carcinomas, although HIF-1 alpha is generally thought to be involved in gastric carcinogenesis [15–17].

Epstein-Barr virus (EBV)-positive gastric carcinomas, a distinct subset of gastric carcinomas, comprise 2~16% of all gastric carcinomas in each country worldwide, irrespective of the national incidence [18]. Several molecular pathways in the pathogenesis of EBV-positive gastric carcinomas are elucidated [19–22], of which, abnormal methylation of multiple genes is one of the crucial initiating mechanisms in EBV-induced carcinogenesis [23, 24]. EBV-encoded latent membrane protein 2A (LMP2A) is a putative viral oncogene that promotes activation of the STAT3, ERK and PI3K/Akt pathways, subsequently leading to tumor cell growth. Additionally, EBV-encoded BARF1 promotes cancer cell proliferation *via* NF-kappaB/cyclin D1 [22].

We evaluated expression of leptin-signaling-related proteins (leptin, leptin-receptor, pSTAT3, ERK, pAkt, mTOR and HIF-1 alpha) and the EBV infection status within cancer cells, and determined their clinicopathological significance in gastric carcinomas.

## Materials and Methods

### Patients

We analyzed 343 cases of gastric carcinoma, which were surgically resected in the Seoul National University Boramae hospital, from 2000 to 2005. Three pathologists (HJL, HK & MSC) reviewed medical records and histopathological findings, such as the Lauren classification [25] and TNM stage based on American Joint Committee on Cancer [26]. The survival data was obtained from the National Statistical Office of Korea, and overall survival was estimated from the date of operation to death or last follow-up visit. The median follow-up period for patient outcome was 77 months (mean: 63.8 months, standard deviation: 42.5).

### Ethics Statement

All human tissue specimens were obtained during diagnostic and therapeutic surgery. The participants did not provide the written consent to participate in the present study. The retrospective study was performed using the stored paraffin blocks containing tissue samples after the pathologic diagnosis, and all of the samples were anonymized before the study. This retrospective study was approved by the Institutional Review Board of Seoul National University Boramae Hospital under the condition of the anonymization (IRB No. 26-2014-13/022, 06-2011-40/106).

### Tissue microarray

After histological review of all tumor sections, one of the representative portions was chosen from the individual donor block and core tissue with 2.0 mm diameter was punched-out by a trephine apparatus. All tissue cores were then inserted in a new recipient block that included 59 tissue cores and one ink core that was used as a direct marker. A total of six tissue array blocks were thus prepared.

### Immunohistochemistry

Immunohistochemistry was performed using an automated immunostainer (Ventana BenchMark XT, Ventana Medical Systems Inc., Tucson, AZ, USA) according to the manufacturer’s protocol. We performed immunohistochemical staining for leptin, leptin- receptor, phosphorylated STAT3 (pSTAT3), ERK, phosphorylated Akt (pAkt), mTOR, and HIF-1 alpha (Table 1). Four pathologists (EC, S-jB, SHK &, MSC,) reviewed the staining results. The expressions of leptin-signaling proteins were sufficiently homogenous in a tumor that one core tissue can be considered to be representative (S1 Fig), and their expressions in gastric carcinoma were upregulated in relation to normal mucosa (S2 Fig).

**Table 1:** Antibodies used for immunohistochemical staining.

Antibody	Expression in tumor cells	Dilution	Source
Leptin (A-20)	cytoplasm	1:50	Santa Cruz, CA, USA
Leptin-receptor (B-3)	membrane	1:25	Santa Cruz
pSTAT3 (D3A7)	nucleus	1:50	Cell Signaling, Danvers, Mass, USA
ERK (D13.14.4E)	nucleus	1:50	Cell Signaling
pAkt (C31E5E)	cytoplasm (or nucleus)	1:50	Cell Signaling
mTOR (49F9)	cytoplasm	1:50	Cell Signaling
HIF-1 alpha (3716S)	cytoplasm (or nucleus)	1:1000	Cell Signaling

Immunohistochemical staining for leptin and leptin-receptor was scored according to the intensity of positively stained tumor cells as follows: negative (score = 0), weak (score = 1), moderate (score = 2), and strong (score = 3). Scores of 1, 2 and 3 were then reclassified as positive.

The following scoring method was applied for mTOR and pAkt: the stained area was categorized as either < 5%, 1; 5–10% = 2, 10–50% = 3, 50–75% = 4, < 75% = 5; and staining intensity was graded as faint = 1, weak = 2, moderate = 3, strong = 4. The stained area was reassorted as: 0,1 = 0; 2, 3 = 1; 4 = 2; 5 = 3 and staining intensity as: 0, 1 = 0; 2 = 1; 3 = 2; 4 = 3; and a total score of 0 to 9 was finally calculated using the percentage of stained area x staining intensity. Subsequently, a score of 0 was categorized as negative, 1–3 as weak, 4–6 as moderate, and 7–9 as strong. Finally, to find statistically significant results, we defined weak, moderate, and strong staining as positive.

We evaluated the results for pSTAT3, ERK, and HIF-1 alpha as positive or negative, because of rare positive staining of tumor cells. The immunostaining results were considered as positive when the percentage of stained tumor cells was > 10% of all tumor cells.

### In situ hybridization (ISH) for EBV-encoded small RNAs

EBV-encoded small RNAs (EBER) were detected by *in situ* hybridization with the EBER detection kit according to the manufacturer’s instructions. Next, the sections were deparaffinized, enzymatically digested with proteinase K, and hybridization solution was applied to the sections, warmed up and incubated. Hybridization detection was performed using a fluorescein-conjugated EBV oligonucleotide probe targeting EBER (Novocastra, Newcastle-upon-Tyne, UK). The slides were then counterstained with Nuclear Fast Red and coverslipped. Positive staining was identified as blue nuclear dots. Nuclear staining in tumor cells was considered positive, and the absence of nuclear staining was considered negative.

### Statistical analysis

The relationship between the clinicopathological features and immunohistochemical staining results was examined by Pearson’s χ^2^ test and Spearman rank correlation coefficient. Patient survival data were obtained by the Kaplan-Meier method and log-rank test. Univariate and multivariate analysis (forward stepwise) were performed by the Cox proportional hazards model. P-values of < 0.05 were considered as statistically significant. All statistical calculations were carried out with SPSS/PC version 21.0 for Windows (IBM SPSS Inc., Chicago, IL, USA).

### Cell culture and small interfering RNA (siRNA) transfection

Three gastric carcinoma cell lines, SNU719, SNU484 and SNU601, which were purchased from the Korean Cell Line Bank (Seoul, Korea), and cells were maintained at 37°C in RPMI 1640 (Gibco BRL, Rockville, MD, USA) supplemented with 10% fetal bovine serum, 2 mmol/L L-glutamine, and antibiotics (100 units/mL penicillin and streptomycin) in a humidified 5% CO_2_/95% air atmosphere. The leptin-receptor-specific siRNA (Ob-R siRNA (h) sc-36116, Santa Cruz, CA, USA) was transfected into gastric carcinoma cells. A scrambled siRNA (Dharmacon, Lafayette, CO, USA) containing a random sequence of nucleotides with no known specificity was used as a negative control. Cells were plated at 50% confluence in 6-well tissue culture plates. After 24 hours, siRNAs were transfected using Lipofectamine 2000 (Invitrogen, Carlsbad, CA, USA) at a final RNA concentration of 50 nM per well. Cells were harvested 72 hours after siRNA transfection.

### Western blot analysis for apoptosis

Cellular extracts from cell lines were prepared by dissociation in lysis buffer, and protein was measured using a BCA protein assay kit. Proteins were resolved by SDS-PAGE using the laemmli buffering system with 15% polyacrylamide running gels and 5% stacking gels, and then transferred to reinforced PVDF membranes (Millipore, Bedford, MA, USA). After blocking with 3% skim milk for 1 hour, the membranes were incubated with primary antibodies against leptin-receptor (sc-8391, 1:500, Santa Cruz,) and caspase-3 (H-227) (sc-7148, 1:1,000, Santa Cruz) for 2 hours at room temperature. After washing, the blots were incubated for 45 minutes at room temperature with a horseradish peroxidase (HRP)-conjugated anti-mouse secondary antibody (Abcam, Cambridge, UK). After extensive washing, the antigen-antibody complexes were visualized by exposing the membranes to X-ray film (Fuji, Japan). An antibody against beta-actin antibody (AC -15, 1:10000, Abcam, Cambridge, UK) was used to verify equal loading and transfer of total proteins.

## Results

### Associations between clinicopathological features and immunohistochemical findings

The overall features of all 343 patients were summarized in Table 2. Based on tumor invasion depth, there were 136 patients (40%) with early gastric carcinoma (EGC) (mucosa or submucosa invasion) and 207 patients (60%) with advanced gastric carcinoma (AGC) (proper muscle or deeper invasion). When grouped by cancer stage (tumor-lymph node-distant metastasis, TNM stage), 144 patients (42%) belonged to the early cancer stage (TNM stage I), and the remaining 199 patients (58%) to the advanced cancer stage (TNM stage II, III or IV).

**Table 2:** Overview of clinicopathological features and immunohistochemical staining results.

	Total	EBV	Leptin	Leptin-receptor	pSTAT3	ERK	pAkt	mTOR	HIF-1α
	(n = 343)	positive	positive	positive	positive	positive	positive	positive	positive
Age				a		a			
<45 years	22 (6%)	3 (14%)	5 (23%)	6 (27%)	1 (5%)	11 (50%)	20 (91%)	12 (55%)	9 (41%)
≥45 years	321 (94%)	29 (9%)	123 (38%)	192 (60%)	28 (9%)	90 (28%)	251 (78%)	157 (49%)	114 (36%)
Sex		a							
male	231 (67%)	28 (12%)	91 (39%)	139 (60%)	19 (8%)	72 (31%)	177 (77%)	112 (48%)	84 (36%)
female	112 (33%)	4 (4%)	37 (33%)	59 (53%)	10 (9%)	29 (26%)	94 (84%)	57 (51%)	39 (35%)
Tumor size			a	a	a	a	a		a
<5cm	179 (52%)	12 (7%)	78 (44%)	94 (53%)	22 (12%)	75 (42%)	154 (86%)	96 (54%)	82 (46%)
≥5cm	164 (48%)	20 (12%)	50 (30%)	104 (63%)	7 (4%)	26 (16%)	117 (71%)	73 (45%)	41 (25%)
Tumor site		a							
low 1/3	194 (57%)	7 (4%)	65 (34%)	117 (60%)	16 (8%)	57 (29%)	151 (78%)	96 (49%)	76 (39%)
middle 1/3	117 (34%)	12 (10%)	52 (44%)	62 (53%)	12 (10%)	41 (35%)	94 (80%)	63 (54%)	41 (35%)
upper 1/3	20 (6%)	12 (60%)	6 (30%)	13 (65%)	1 (5%)	1 (5%)	16 (80%)	5 (25%)	4 (20%)
whole	12 (3%)	1 (8%)	5 (42%)	6 (50%)	0	2 (17%)	10 (83%)	5 (42%)	2 (17%)
Lauren		a	a	a				a	
intestinal	160 (47%)	4 (3%)	78 (49%)	106 (66%)	17 (11%)	49 (31%)	130 (81%)	92 (58%)	65 (41%)
diffuse	139 (41%)	25 (18%)	32 (23%)	65 (47%)	8 (6%)	40 (29%)	105 (76%)	56 (40%)	45 (32%)
mixed	44 (13%)	3 (7%)	18 (41%)	27 (61%)	4 (9%)	12 (27%)	36 (82%)	21 (48%)	13 (30%)
Invasion depth		a	a	a	a	a	a		a
mucosa	70 (20%)	0	33 (47%)	26 (37%)	8 (11%)	36 (51%)	60 (86%)	36 (51%)	37 (53%)
submucosa	66 (19%)	3 (5%)	28 (42%)	44 (67%)	11 (17%)	30 (45%)	59 (89%)	44 (67%)	37 (56%)
proper muscle	37 (11%)	7 (19%)	12 (32%)	22 (59%)	5 (14%)	11 (30%)	30 (81%)	17 (46%)	13 (35%)
subserosa	76 (22%)	13 (17%)	27 (36%)	49 (64%)	2 (3%)	9 (12%)	61 (80%)	26 (34%)	10 (13%)
serosa, adjacent structure	94 (27%)	9 (10%)	28 (30%)	57 (61%)	3 (3%)	15 (16%)	61 (65%)	46 (49%)	26 (28%)
Lymph node			a	a	a	a	a		a
no metastasis	183 (53%)	12 (7%)	80 (44%)	92 (50%)	21 (11%)	77 (42%)	156 (85%)	92 (50%)	84 (46%)
metastasis	160 (47%)	20 (13%)	48 (30%)	106 (66%)	8 (5%)	24 (15%)	115 (72%)	77 (48%)	39 (24%)
TNM stage		a	a		a	a	a		a
IA	120 (35%)	3 (3%)	56 (47%)	61 (51%)	18 (15%)	58 (48%)	105 (88%)	69 (58%)	69 (58%)
IB	24 (7%)	3 (13%)	13 (54%)	14 (58%)	3 (13%)	8 (33%)	20 (83%)	10 (42%)	9 (38%)
IIA	37 (11%)	6 (16%)	12 (32%)	23 (62%)	1 (3%)	12 (32%)	33 (89%)	16 (43%)	9 (24%)
IIB	31 (9%)	7 (23%)	8 (26%)	16 (52%)	0	2 (6%)	19 (61%)	12 (39%)	4 (13%)
IIIA	29 (8%)	3 (10%)	7 (24%)	20 (69%)	3 (10%)	4 (14%)	22 (76%)	14 (48%)	10 (34%)
IIIB	34 (10%)	4 (12%)	11 (32%)	24 (71%)	2 (6%)	6 (18%)	28 (82%)	13 (38%)	1 (3%)
IIIC	56 (16%)	4 (7%)	15 (27%)	31 (55%)	1 (2%)	8 (14%)	35 (63%)	28 (50%)	20 (36%)
IV	12 (3%)	2 (17%)	6 (50%)	9 (75%)	1 (8%)	3 (25%)	9 (75%)	7 (58%)	1 (8%)

^a^ *P* < 0.05. EBV, Epstein-Barr virus.

**Fig 1:**
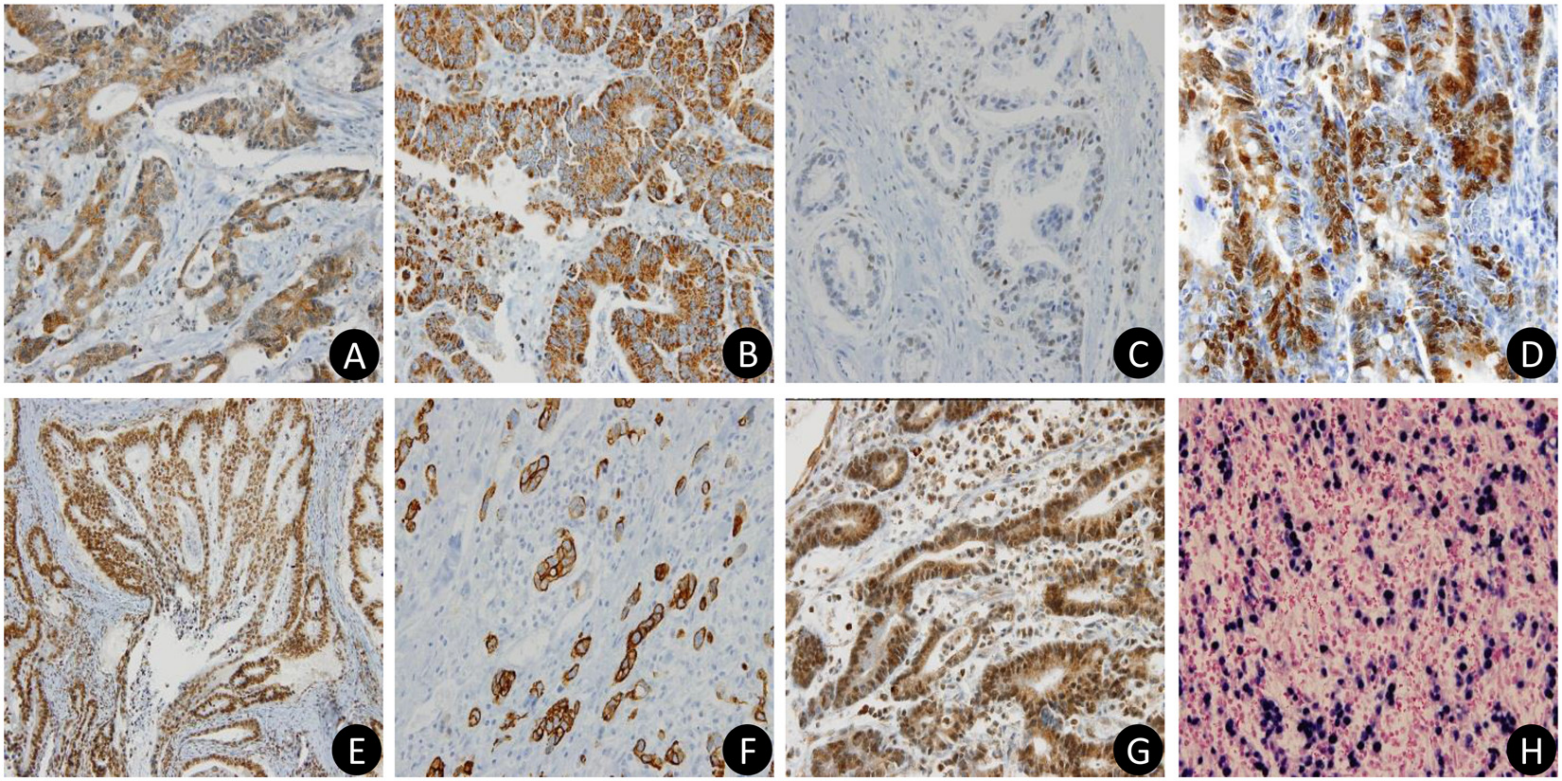
Representative immunohistochemical features of seven protein markers including proteins related to leptin signaling pathway (A-G) and *in situ* hybridization of Epstein-Barr virus encoded small RNAs (H). (**A**) Leptin shows cytoplasmic expression in cancer cells (x 400). (**B**) Leptin-receptor exhibits membranous staining in cancer cells (x 200). (**C**) pSTAT3 shows nuclear staining in cancer cells (x 400). (**D**) ERK has nuclear positivity in cancer cells (x 400). (**E**) pAkt shows cytoplasmic or nuclear staining in cancer cells (x 200). (**F**) mTOR has cytoplasmic positivity in cancer cells (x 200). (**G**) HIF-1 alpha has cytoplasmic or nuclear staining in cancer cells (x 400). (**H**) Most of the cancer cells show blue colored nuclear staining on *in situ* hybridization for Epstein-Barr virus (x 400).

Expression of leptin and leptin-receptor were observed in 37% (128/343) and 58% (198/343) of total gastric carcinomas, respectively. Leptin signaling-related proteins were commonly expressed in gastric carcinomas except for pSTAT3; pAkt positive in 79% (271/343) of gastric carcinomas, mTOR in 49% (169/33), HIF-1 alpha in 36% (123/343), ERK in 29% (101/343), and pSTAT3 in 8% (29/343). The representative immunohistochemical staining patterns were shown in Fig 1.

The relationship between clinicopathological findings and expressions were as follows: expression of leptin, pSTAT3, ERK, pAkt and HIF-1 alpha were more often positive in the EGC group than in AGC (*P* < 0.05). Expression of leptin, pSTAT3, ERK, pAkt and HIF-1 alpha were correlated with the absence of lymph node metastasis, while leptin-receptor positivity was related with lymph node metastasis (*P* < 0.05, respectively). Accordingly, each expression of leptin, pSTAT3, ERK, pAkt, and HIF-1alpha showed a correlation with the early cancer stage (TNM stage based on tumor invasion depth-lymph node metastasis-distant metastasis) (*P* < 0.05, respectively). Leptin, leptin-receptor and mTOR expression were more likely to be found in the Lauren intestinal type than in diffuse type (*P* < 0.05, respectively). Leptin-receptor expression showed a positive correlation with a larger tumor size (≥ 5cm) (*P* < 0.05).

### Clinicopathological characteristics of EBV-positive gastric carcinomas

There were 32 cases of the total 343 gastric carcinomas (9%) in the EBV-positive group. The EBV-positive group revealed a male predominance, a predilection of tumor location in the upper 1/3 of the stomach, more frequent diffuse Lauren subtype and more advanced cancer stage (TNM stage) (*P* < 0.05, respectively) (Tables 2 & 3). In terms of leptin signaling-related protein, the EBV-positive tumors have a more abundant expression of leptin-receptor and a lower expression of mTOR compared to non EBV-related tumors, but a putative correlation with EBV cannot be shown in this way (Table 3).

**Table 3:** Comparison of immunohistochemical features between EBV-positive gastric carcinomas and EBV-negative gastric carcinomas.

	EBV-negative	EBV-positive	
	(n = 311)	(n = 32)	*P value*
Age (years)			
< 45 years	19 (6%)	3 (9%)	
≥45 years	292 (94%)	29 (91%)	NS
Sex			
male	203 (65%)	28 (87%)	
female	108 (35%)	4 (13%)	0.011
Tumor location			
low 1/3	187 (60%)	7 (22%)	
middle 1/3	105 (33%)	12 (38%)	
upper 1/3	8 (3%)	12 (38%)	
whole	11 (4%)	1 (2%)	< 0.001
Lauren			
intestinal	156 (50%)	4 (13%)	
diffuse	114 (37%)	25 (78%)	
mixed	41 (13%)	3 (9%)	< 0.001
Cancer stage			
earlier (TNM Stage I)	138 (44%)	6 (19%)	
advanced (TNM Stage II, III, IV)	173 (56%)	26 (81%)	0.005
Leptin			
negative	197 (63%)	18 (56%)	
positive	114 (37%)	14 (44%)	NS
Leptin-receptor			
negative	137 (44%)	8 (25%)	
positive	174 (56%)	24 (75%)	0.038
pSTAT3			
negative	284 (91%)	30 (94%)	
positive	27 (9%)	2 (6%)	NS
ERK			
negative	216 (69%)	26 (81%)	
positive	95 (31%)	6 (19%)	NS
pAkt			
negative	66 (21%)	6 (19%)	
positive	245 (79%)	26 (81%)	NS
mTOR			
negative	149 (48%)	25 (78%)	
positive	162 (52%)	7 (22%)	0.001
HIF-1α			
negative	195 (63%)	25 (78%)	
positive	116 (37%)	7 (22%)	NS

EBV, Epstein-Barr virus; NS, not significant.

### Different prognostic implication of leptin signaling-related proteins according to clinicopathological subsets of gastric carcinomas

**Fig 2:**
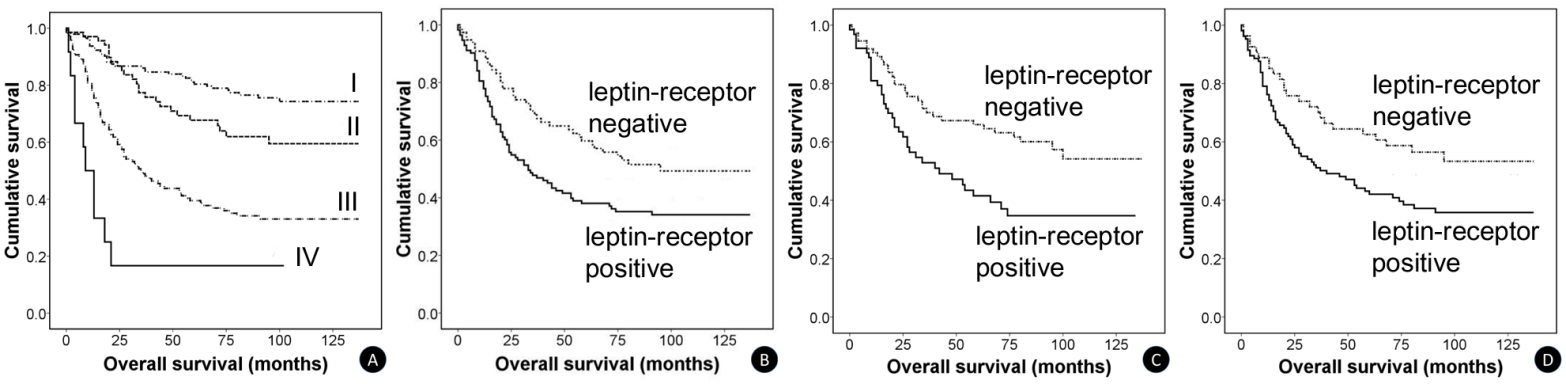
Kaplan-Meier survival curves in each group of gastric carcinomas according to leptin-receptor expression status (A-D). (**A**) Advanced TNM stage was associated with poor survival of patients in all cases of gastric carcinoma (n = 343) (*P* < 0.001). (**B**) Leptin-receptor positivity was related with unfavorable survival outcome in the advanced gastric carcinoma subgroup (n = 207) (*P* = 0.015). (**C**) Leptin-receptor positivity was correlated with poor survival rate in the diffuse-type gastric carcinoma subgroup (n = 139) (*P* = 0.007). (**D**) Leptin-receptor positivity was compatible with lower survival rate in patients with lymph node metastasis (n = 160) (*P* = 0.023).

Patient outcome (median follow-up period: 77 months) was mortality in 148 of the total 343 (43%) patients, which included 10 of the 32 EBV-positive group (31%) patients. pTNM stage was the absolute independent prognostic factor, on univariate and multivariate analyses in all 343 patients (*P* < 0.05) (Fig 2A). None of the examined protein markers (leptin, leptin- receptor, pSTAT3, ERK, pAkt, mTOR and HIF-1 alpha) alone, had a statistical significance on overall survival in all 343 patients on multivariate analysis.

We classified all 343 gastric carcinomas based on clinicopathological criteria, to identify the correlation between expression of the examined markers and patient survival according to subsets of gastric carcinomas. All patients were initially categorized into two groups based upon tumor invasion depth; early gastric cancer (EGC: tumor limited to the mucosal and submucosal invasion) and advanced gastric cancer (AGC). There were 136 cases of EGC and 207 cases of AGC. In 207 advanced gastric carcinomas, leptin-receptor expression was correlated with a poor survival of patients in univariate and multivariate analyses (*P* < 0.05) (Fig 2B). Furthermore, the EBV-positive AGC subgroup (n = 29) tended to have better patient survival than the EBV-negative AGC subgroup (n = 178), but there was no statistical significance (*P* = 0.06).

All patients were divided into intestinal, diffuse or mixed subtype according to the Lauren classification based on microscopic histopathology. There were 139 (41%) cases of diffuse type, 160 (47%) cases of intestinal type, and 44 (13%) cases of mixed type. Leptin-receptor positivity in the Lauren diffuse type was correlated with a poor survival of patients in univariate and multivariate analysis (*P* < 0.05) (Fig 2C). Additionally, leptin-receptor expression was compatible with poor survival outcome in 160 (47%) patients with lymph node metastasis, on univariate and multivariate analysis (*P* < 0.05) (Fig 2D).

Expression of pAkt in 32 (9% = 32/343) cases of EBV-positive gastric carcinomas appeared to be correlated with better survival of patients on univariate analysis, but lost significance on multivariate analysis.

### Effect by leptin-receptor inhibition in stomach cancer cells

**Fig 3:**
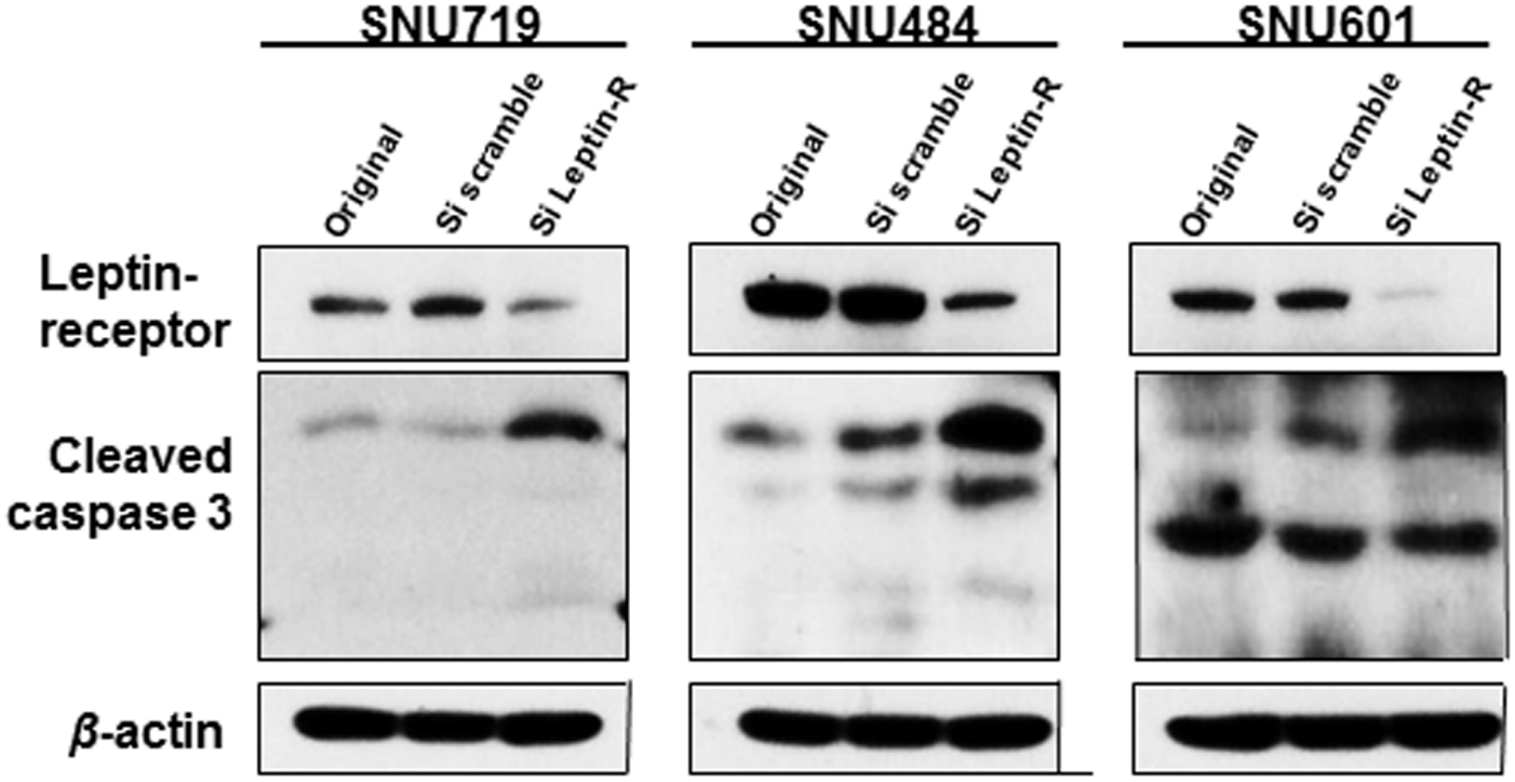
Effect of leptin-receptor inhibition in gastric carcinoma cells. Leptin-receptor in total cell lysate was inhibited by siRNA against leptin-receptor (50 μM). Cleaved caspase 3 (p11, p17 and p20 subunits) was remarkably increased in gastric carcinoma cell lines transfected with siRNA against leptin-receptor, comparing to each original cell line and each scrambled siRNA-treated cell line. The beta-actin was loading controls. ‘Si scramble’ means cells transfected with scrambled siRNA, and ‘Si Leptin-R,’ cells transfected with siRNA against leptin-receptor.

In three gastric carcinoma cell lines, transfection of siRNA against leptin-receptor resulted in a remarkable increase of cleaved caspase 3 (p11, p17 and p20 subunits), comparing to each original cells and each scrambled siRNA-transfected cells (Fig 3). In other words, leptin-receptor is suggested to prevent apoptosis in gastric carcinoma cells.

## Discussion

The present study highlighted that leptin-receptor expression can be an independent poor prognostic factor in specific subsets of gastric carcinomas. Expression of leptin-receptor was correlated with adverse clinicopathological parameters, including lymph node metastasis and Lauren diffuse subtype. Leptin-receptor positivity was related with poor survival of patients, especially in the AGC group, Lauren diffuse type group, and lymph node metastasis group, on univariate and multivariate analyses (*P* < 0.05). Our results were consistent with the report of Ishikawa *et al* that lymph node metastasis and distant metastasis were frequently observed in the leptin-receptor positive group among gastric carcinoma patients [27]. As one of explanations for that leptin-receptor expression group showed poor survival rate of patients, we suggest that leptin-receptor counteracts apoptosis in cancer cells, since leptin-receptor inhibition induced an increased apoptosis in the present study. Antiapoptotic effect of leptin-receptor has been described in other human cancers [28]. Leptin-receptor may thus play a pivotal role in the progression of gastric carcinoma. However, to the best of our knowledge, apart from the study of Ishikawa *et al*, there have been few studies on the prognostic significance of the leptin-receptor in gastric carcinoma. Therefore, further studies are required to clearly define the prognostic value of leptin-receptor expression in gastric carcinomas.

The present study indicated that leptin signaling proteins may contribute to HIF-1 alpha-mediated angiogenesis in gastric carcinoma. HIF-1 alpha expression was positively correlated with the expression of pSTAT3, ERK, pAkt and mTOR (*P* < 0.05). This result was consistent with previous reports of a positive interrelationship between molecules involved in leptin-signaling and HIF-1 alpha in gastric carcinoma [29] and renal cell carcinoma [30].

The present study determined the clinicopathological characteristics of EBV-positive gastric carcinomas. We found that the EBV-infection rate in gastric carcinomas was 9%; EBV-positive gastric carcinomas were more prevalent in male patients; tumor location in the upper 1/3 stomach; and the Lauren diffuse type. These results reiterated the findings of previous reports [18, 22, 31–34]. EBV-infection may have only limited prognostic value, at most, only in the advanced gastric cancer group. There was a tendency that EBV-positive AGC subgroup had a better patient survival compared with EBV-negative AGC subgroup (*P* = 0.06). There are different opinions on EBV-infection as a prognostic factor in gastric carcinomas. Most authors suggest that EBV is not an independent prognostic factor [32–34]. On the other hand, some authors claim that EBV is a good prognostic factor for survival of patients with gastric carcinomas through meta-analysis [35]. Global methylation of genes is known as a basic and initial mechanism in EBV-associated carcinogenesis [23], and multistep alteration of genes occur subsequently. Therefore, EBV-infection itself may not act as an independent prognostic factor. While leptin-receptor expression was more frequently observed in EBV-positive subgroup than in EBV-negative subgroup, mTOR was less expressed in EBV-positive subgroup than in EBV–negative subgroup (*P* < 0.05, respectively). Moreover, leptin expression was quite less observed in EBV-positive group. Possibly, leptin signaling may not be associated with EBV-induced carcinogenesis. Surprisingly, expression of pAkt was related with a good survival of patients with 32 EBV-positive gastric carcinomas (*P* < 0.05). Previous reports have shown that expression of pAkt correlates with poor prognosis in conventional gastric carcinoma [36–39]. We are unaware of any study that analyzes the prognostic impact of pAkt limited to EBV-positive gastric carcinoma cases. The analysis of more EBV-positive gastric carcinoma cases is needed to clarify the association between pAkt expression and prognosis of EBV-positive gastric carcinoma. Besides, our research corroborated a previous study by Sukawa *et al* that showed a lack of statistical difference in pAkt expression between EBV-positive gastric carcinoma and EBV-negative gastric carcinoma.

In conclusion, leptin signaling-related proteins and EBV status show different significance on patient survival according to subsets of gastric carcinoma. Expression of leptin-receptor can be an independent poor prognostic indicator in the advanced gastric cancer group, Lauren diffuse group, and the lymph node metastasis group. EBV-positive may have a limited prognostic value only in the advanced gastric cancer group, and leptin signaling may not be involved in EBV-positive gastric carcinogenesis. Further studies are required to understand how leptin signaling-related proteins and the EBV status play a role in regulating carcinogenesis, and ultimately to establish them as therapeutic targets in gastric carcinomas.

## Supporting Information

S1 Fig Immunohistochemical staining patterns for leptin signaling-related proteins and in situ hybridization for Epstein-Barr virus-encoded small RNAs shown in full tissue sections of gastric carcinoma. The expressions of leptin-signaling proteins and in situ hybridization for EBV-encoded small RNAs are fairly or thoroughly homogenous in a tumor (x 40).
(TIF)

S2 Fig Immunohistochemical features of leptin signaling-related proteins in normal gastric mucosa. Normal gastric mucosa reveals no staining or vaguely week staining (x 40).
(TIF)
