# Supplementary figures and images for: Implication of Leptin-Signaling Proteins and Epstein-Barr Virus in Gastric Carcinomas
Source: PLoS One. 2015 Jul 6;10(7):e0130839. doi: 10.1371/journal.pone.0130839 (PMC4493019; doi:10.1371/journal.pone.0130839)

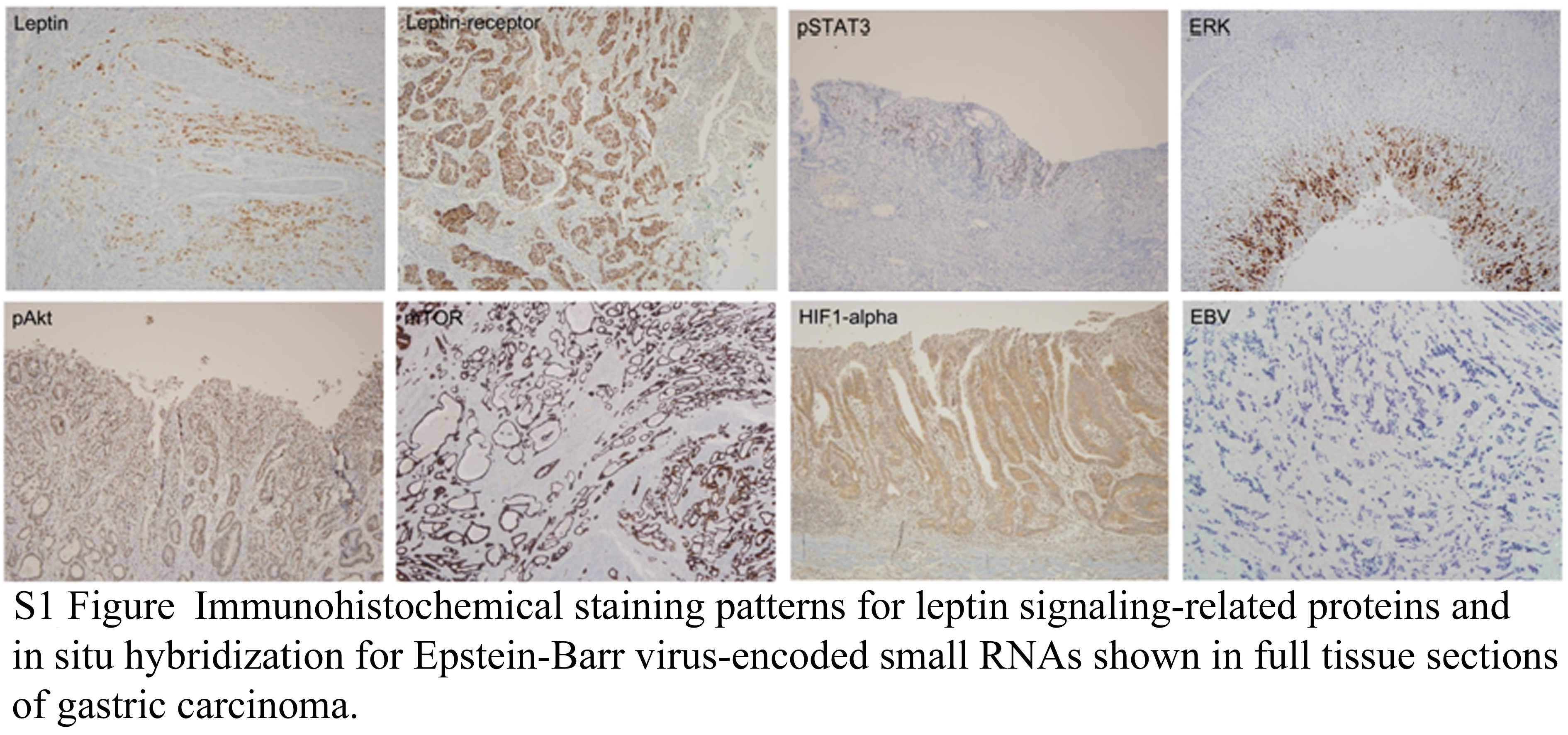

Supplement: S1 Fig — The expressions of leptin-signaling proteins and in situ hybridization for EBV-encoded small RNAs are fairly or thoroughly homogenous in a tumor (x 40). (TIF) [file pone.0130839.s001.tif]

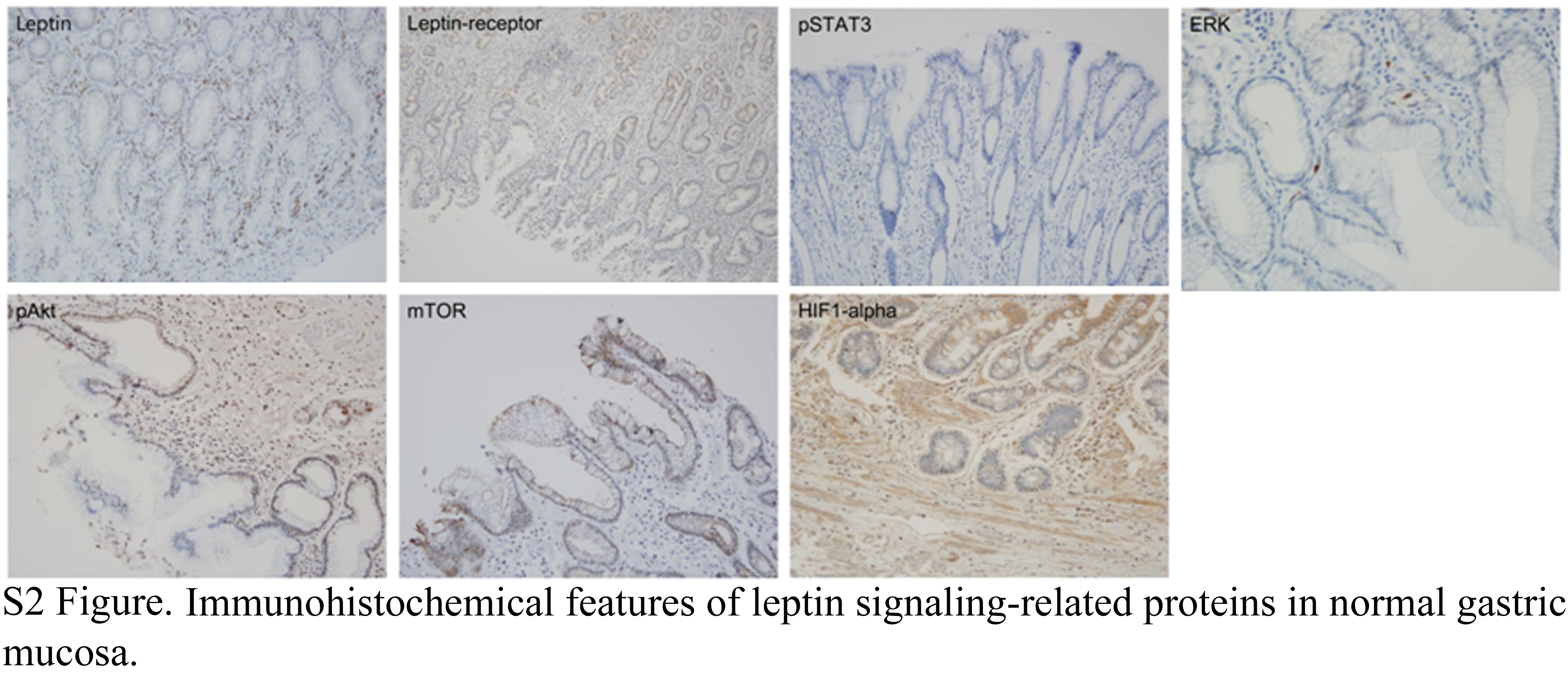

Supplement: S2 Fig — Normal gastric mucosa reveals no staining or vaguely week staining (x 40). (TIF) [file pone.0130839.s002.tif]
